# Aerobic Exercise Attenuates Pain Sensitivity: An Event-Related Potential Study

**DOI:** 10.3389/fnins.2021.735470

**Published:** 2021-09-21

**Authors:** Kangyong Zheng, Changcheng Chen, Suyong Yang, Xueqiang Wang

**Affiliations:** ^1^Department of Sport Rehabilitation, Shanghai University of Sport, Shanghai, China; ^2^Department of Rehabilitation Medicine, Qingtian People’s Hospital, Zhejiang, China; ^3^School of Psychology, Shanghai University of Sport, Shanghai, China; ^4^Department of Rehabilitation Medicine, Shanghai Shangti Orthopaedic Hospital, Shanghai, China

**Keywords:** aerobic exercise, hypoalgesia, event-related potential, oscillation, exercise intensity

## Abstract

In this study, electroencephalography (EEG) was utilized to explore the neurophysiological mechanisms of aerobic exercise-induced hypoalgesia (EIH) and provide a theoretical basis for the application of aerobic exercise in pain assessment and treatment. Forty-five healthy subjects were randomly divided into moderate-intensity aerobic exercise [70% heart rate reserve (HRR)], low-intensity aerobic exercise (50% HRR), or control groups (sitting). Aerobic exercise was performed with cycling. Pressure pain threshold (PPT), heat pain threshold (HPT), event-related potential (ERP) induced by contact heat stimulus and pain scoring were measured before and after the intervention. We found that moderate-intensity aerobic exercise can increase the PPT (rectus femoris: *t* = −2.71, *p* = 0.017; tibialis anterior muscle: *t* = −2.36, *p* = 0.033) and HPT (tibialis anterior muscle: *t* = −2.219, *p* = 0.044) of proximal intervention sites rather than distal sites, and decreased pain scorings of contact heat stimulus. After moderate-intensity aerobic exercise, alpha oscillation power reflecting the central descending inhibitory function was enhanced (*t* = −2.31, *p* < 0.05). Low-intensity aerobic exercise mainly reduced the pain unpleasantness rating (Block 1: *t* = 2.415, *p* = 0.030; Block 2: *t* = 3.287, *p* = 0.005; Block 4: *t* = 2.646, *p* = 0.019; Block 5: *t* = 2.567, *p* = 0.022). Aerobic exercise had an overall EIH effect. Its hypoalgesic effect was related to exercise intensity and affected by the site and type of pain stimulus. Moderate-intensity aerobic exercise effectively reduced the sensitivity to various painful stimuli, and low-intensity aerobic exercise selectively inhibited the negative emotional pain response. The hypoalgesic mechanism of aerobic exercise involves the enhancement of the central descending inhibitory function.

## Introduction

As a non-drug treatment, exercise has been widely used for chronic pain management (Brosseau et al., 2008; van Middelkoop et al., 2010; Chen et al., 2012; Loew et al., 2012; Steele et al., 2020). Exercise reduces the sensitivity to noxious stimuli through a process called exercise-induced hypoalgesia (EIH), which is an endogenous pain regulation (Thorén et al., 1990). EIH is manifested as an increased pain perception threshold and pain tolerance, and a change in pain ratings during or after exercise (Koltyn, 2000).

Existing research has shown that three types of exercise produce EIH effects, and these types are aerobic, dynamic resistance, and isometric contraction exercise (Naugle et al., 2012). Compared with the two other types of exercise, aerobic exercise is more diversified and is thus conducive to personalized exercise programs. However, the factors that affect aerobic EIH are complex; in particular, the influence of exercise intensity remains unclear (Polaski et al., 2019). Early studies have reported that exercise intensity must exceed 70% maximal oxygen uptake [VO (2) max], and as the intensity increases, the effect of aerobic EIH increases further (Kemppainen et al., 1990). The dependence of aerobic EIH effects on high intensity may limit the clinical application of aerobic exercise (Naugle et al., 2012). Given that pain is a dynamic process of physiology and psychology, accurately assessing the pain sensitivity is difficult. Therefore, using accurate sensory assessment methods is conducive to identifying the aerobic EIH effect. Recently, some researchers provided evidence that aerobic exercise intensities of 70% HRR and 50% HRR reduce pain ratings, especially the former for a greater dose–response effect (i.e., the more intense exercise produces larger effects) (Naugle et al., 2014).

Researchers have recently discovered that the aerobic EIH effect also has a clinical evaluation value. In some patients with chronic musculoskeletal pain, the effect of aerobic EIH is weakened and even reversed (i.e., increased pain sensitivity) for potential mechanisms of abnormal descending inhibition or excessive activation of muscle nociceptive afferents (Vierck et al., 2001; Staud et al., 2005; Naugle et al., 2012). High pain sensitivity is an important feature and risk factor of chronic pain (Meints et al., 2019; Nahman-Averbuch et al., 2019). In addition, the EIH efficiency before total knee arthroplasty is positively correlated with pain relief after surgery (Vaegter et al., 2017a). Therefore, evaluations of EIH are helpful in monitoring and evaluating the endogenous pain regulation system function (Gomolka et al., 2019).

Currently, the exercise programs concerning aerobic EIH have contained various exercise types (e.g., cycling, running), pain induction techniques (e.g., pressure and heat stimuli) and measurements [e.g., functional magnetic resonance imaging, event-related potential (ERP)] (Janal et al., 1984; Scheef et al., 2012; Jones et al., 2016; Vaegter et al., 2017b). Methodological differences among studies make it difficult to identify the effect of exercise on different pain aspects (Naugle et al., 2012, 2014; Jones et al., 2019). For example, it was reported that parameters of the exercise, like types, durations, and intensities of exercise, may determine which system is activated (Naugle et al., 2012).

Given that the exact neural mechanisms are still unclear, some studies have been proposed to elucidate EIH (Micalos et al., 2014; Jones et al., 2016). A recent study evaluated the impact of cycling on brain activation to pain in fibromyalgia and found that cycling seemed to activate brain areas, like the left dorsalateral prefrontal lobe of the anterior insula, which involved in descending pain inhibition, decreasing pain sensitivity (Ellingson et al., 2016). A novel ERP study found that the effect of exercise on the amplitude of somatosensory and laser evoked potentials was negligible when compared to that of controlled condition, yet failing to identify the straightforward mechanism of EIH (Jones et al., 2016). Perhaps a potential mechanism is the activation of endogenous opioid system, which links to changes in pain sensitivity (Naugle et al., 2012). Additionally, a plausible basis for EIH is by a functional restoration of the descending pain-inhibition pathways and/or desensitization (Naugle and Riley, 2014; Micalos et al., 2015). However, human research has not provided consistent evidences (Naugle et al., 2012). Further research should be conducted on the hypoalgesia effect and central nervous system mechanisms of moderate- and low-intensity aerobic exercise to promote the application of aerobic exercise in pain management.

This study evaluated the hypoalgesic effect of aerobic exercise among healthy subjects. The main research objectives are to (i) explore the influence of low- and moderate-intensity aerobic exercise on pain sensitivity and (ii) examine the neurophysiological mechanisms of aerobic EIH.

## Materials and Methods

### Participants

Forty-five healthy subjects (23 males and 22 females) were recruited online and via posters. The average age of the subjects was 24.47 years old. The inclusion criteria were as follows: 18–35 years old; right-handed; without non-persistent or intermittent pain in the last 3 months; no smoking and drinking habits; good health; non-professional athlete. The exclusion criteria were as follows: has participated in the same type of experiment in the past week; women who are menstruating; has a cardiovascular disease, such as hypertension, arrhythmia, and cardiomyopathy; has a history of neurological diseases, such as epilepsy, cerebral palsy, and spinal cord injury; has a disease that affects the musculoskeletal system, such as arthritis, tendonitis, and disc herniation; long-term use of drugs that affect the nervous and musculoskeletal systems; and presence of other acute symptoms, such as colds, fever, and cough, on the day of the experiment.

This study was approved by the Ethics Committee of Shanghai University of Sport. All subjects signed an informed consent form before the test. Meanwhile, demographics and clinical characteristics of the subjects were collected, including age, height, weight, grip strength, body mass index (BMI), sex ratio, years of education, and physical activity levels. Physical activity levels were assessed with the International Physical Activity Questionnaire–Short (IPSQ-S) which is a widely used self-reporting questionnaire for assessing physical activity (van Poppel et al., 2010; Lee et al., 2011). For this questionnaire, subjects were asked about the weekly frequency of walking, moderate intensity and high intensity activities in the past 7 days and the cumulative time per day, gaining an overall estimate of physical activity (Craig et al., 2003).

### Interventions

**FIGURE 1:**
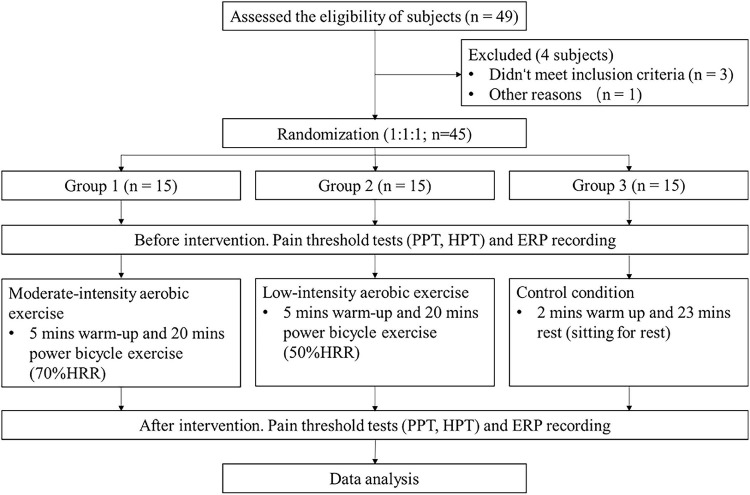
Experimental flow chart. PPT, pressure pain threshold; HPT, heat pain threshold; CHEPs, contact heat-evoked potentials; HRR, heart rate reserve.

This study was conducted in the psychological experiment center of Shanghai University of Sport. The experimental process is shown in Figure 1. Forty-five healthy subjects were recruited and randomly (1:1:1) divided into three groups via computer generated sequence, namely, moderate-intensity exercise group (Group 1), low-intensity exercise group (Group 2), and control group (Group 3). Aerobic exercise was individually performed in the form of cycling (Monark, Switzerland), and an H10 Polar heart rate belt (Polar, Finland) was used to measure the rest and exercise heart rates. Groups 1 and 2 received a power bicycle exercise intervention for 25 min (5 min of warm-up and 20 min of training). The intervention time for Group 3 was also 25 min (2 min of warm up and 23 min of rest). The exercise intensity for Groups 1–3 was 70% HRR, 50% HRR, and sitting for rest, respectively (American College of Sports Medicine Position Stand, 1998; Duncan et al., 2013). The subjects were instructed to report their rating of perceived exertion (RPE) scores, and their heart rates were recorded every 5 min. A Borg 6–20 RPE scale, a subjective rating of the intensity of exercise based on the feeling of exertion, ranging from 6 “no exertion at all” to 20 “maximal exertion,” was clearly visible to the subjects during the exercise (Williams, 2017).

### Test Methods

The three groups of subjects were tested for pressure pain threshold (PPT), heat pain threshold (HPT), and ERP induced by contact heat stimulus before and after the intervention.

#### Pressure Pain Threshold Test Method

PPT was tested using a pressure algometer (Wagner FPX, United States) with a probe diameter of 1.0 cm that exerts pressure up to 20 kg at a rate of 0.5 kg/s (Gerhardt et al., 2017). Four test sites on the right side were tibialis anterior muscle (one-half of the distance between the tibial tuberosity and lateral malleolus), rectus femoris (10 cm above the upper margin of the patella), sacrospinalis (2 cm away from the spinous process of lumbar 3), and thenar muscle (the thenar eminence of the hand). Tibialis anterior, one of the most important muscles during bicycling, did not change moving range dramatically during bicycling, allowing stable and reliable pain assessments (Blake et al., 2012; Jobson et al., 2013; Flouris et al., 2015; Jongerius et al., 2021). Three marks were placed approximately 1 inch apart over test muscle to ensure similar test point before and after inventions. As soon as the subject felt pain and said “pain”, the tester stopped pressing immediately and the value displayed on the algometer was PPT. The test was performed three times for each site with a time gap of 10 seconds, and the average value was obtained.

#### Heat Pain Threshold Test Method

HPT test was performed on three sites on the right side by using the PATHWAY sensory evaluation system (Medoc Ltd., Israel). The three sites were the forearm (5 cm above the volar wrist transverse striation), rectus femoris (10 cm above the upper margin of the patella), and tibialis anterior muscle (one-half of the distance between the tibial tuberosity and lateral malleolus). Three marks were placed approximately 1 inch apart over each test muscle to ensure similar test point before and after inventions. A slow-heating probe (AST stimulator) was used at a baseline temperature of 36 °C, heating rate of 1 °C/s and cooling rate of 3 °C/s. HPT was determined as the temperature when subjects initially felt pain from the rising temperature and pressed a button to terminate the rise. The test was performed three times for each site with a time gap of 5 s, and the average value was obtained.

#### Contact Heat Stimulus Parameter Setting and Pain Scoring

A PATHWAY sensory evaluation system with a fast-heating probe was used. The stimulation intensity was 50°C the heating speed was 70°C/s, and the cooling speed was 40°C/s (Meng et al., 2013). The stimulation sites were tibialis anterior muscles (one-half of the distance between the tibial tuberosity and lateral malleolus) of both legs. The interval between two stimuli was 18–20 s, and 5 stimuli formed a block. After a block ended, switched to the opposite side, alternating left and right. Each subject received contact heat stimuli in 6 blocks and 30 stimuli in total. About 3 s after each stimulation, the subjects were instructed to orally report pain intensity (no pain—worst pain imaginable) and unpleasantness (no unpleasantness—most unpleasantness) on numerical rating scale (NRS) that ranged from 0 to 10. The pain intensity and pain unpleasantness of each block were averaged, respectively, and the average values were obtained (Li et al., 2020).

### Electroencephalography Data Collection and Processing

#### Electroencephalography Data Collection

The Brain Products system (BP, Germany) with a band pass filter of 0.01–100 Hz and sampling frequency of 1,000 Hz was used for data collection. EEG data were recorded by 64 Ag–AgCl scalp electrodes placed in accordance with the International 10–20 System. FCz was the reference (Lei and Liao, 2017), and the ground electrode was AFz. Eye blinks and ocular movement signals were recorded with a vertical electrooculographic electrode placed 1 cm below the lower eyelid. The electrode impedances did not exceed 10 kΩ.

#### Electroencephalography Data Preprocessing

The BP Analyzer 2.1 software was used to preprocess the EEG data. First, the data were re-referenced to TP9/TP10. S, 1–30 Hz band-pass filtering was performed. Third, eye blink and movement signals were removed through independent component analysis. Fourth, the EEG data were segmented into epochs with a time window of 1,500 ms (ranging from 500 ms pre-stimulus to 1,000 ms post-stimulus), and baseline correction was performed with the pre-stimulus interval (–500 ms to 0 ms) (Hu et al., 2014). Lastly, Epochs exceeding ± 80 μV were rejected as considered contaminated by artifacts.

#### Time-Domain Analysis

For ERP, the peak latencies and amplitudes of N2 and P2 waves were detected from the average waveform recorded by Cz electrode (Wydenkeller et al., 2008; Jones et al., 2016, 2019). The time for contact heat stimulus to reach the target temperature was about 200 ms. N2 and P2 waves were defined as the most negative and positive deflections between 350 and 700 ms after stimulus onset, respectively (Tu et al., 2016; Jin et al., 2018).

#### Frequency-Domain Analysis of Pre-stimulus Event-Related Potential Signal Oscillation

Fast Fourier transform (FFT) was applied to the ERP time domain signals before the stimulus to explore the influence of exercise on nerve oscillation at different frequencies. The procedure yielded an ERP spectrum ranging from 1 Hz to 30 Hz. Individual- and group-level ERP spectra were obtained after superposing and averaging.

### Statistical Analysis

One-way ANOVA was applied for inter-group comparison of PPT, HPT and ERP results of pretest, posttest and pre–post differences (values before minus after intervention), respectively; and paired *T*-test was used for intra-group comparison. For the pain intensity and pain unpleasantness ratings, test time (pretest, posttest) × block (Blocks 1–6) × intervention condition (moderate-intensity exercise, low-intensity exercise, and control) were analyzed through repeated-measure ANOVA. Greenhouse-Geisser correction was applied when the spherical test was not met (Lo et al., 2019). Spearman correlation analysis was conducted on the three types of exercise intensity index (RPE score, exercise heart rate, and %HRR) with a difference in the various pain indicators before and after intervention. SPSS 22.0 was applied for statistical analysis, and *p* < 0.05 was considered statistically significant.

## Results

### Demographics and Clinical Characteristics and Exercise Intensity Monitoring

The forty-five subjects were randomly divided into three groups at a ratio of 1:1:1. Demographics and clinical characteristics, such as age, height, weight, resting heart rate, and physical activity level, showed no statistical difference among the groups (*p* > 0.05; Table 1). The differences in the exercise intensity monitoring results among groups, including heart rate,%HRR and RPE score during exercise, were all statistically significant (*p* < 0.001); that is, the highest values of heart rate,%HRR and RPE score were found in the moderate-intensity exercise group, and the lowest ones existed in control group.

**TABLE 1:** Demographics and clinical characteristics and exercise intensity monitoring of the subjects.

	**Group 1 (*n* = 15)**	**Group 2 (*n* = 15)**	**Group 3 (*n* = 15)**	**F**	***p***
Age (Y)	24.733.17	24.402.16	24.273.01	0.109	0.897
Height (cm)	167.207.72	167.8010.71	168.2710.89	0.044	0.957
Weight (kg)	59.339.77	62.9010.28	60.1012.32	0.449	0.641
Grip strength (kg)	35.4310.91	37.5812.68	37.2612.50	0.133	0.876
BMI (kg/m^2^)	21.132.40	22.211.70	21.012.05	1.521	0.23
Sex ratio (male/female)	8/7	7/8	8/7		>0.05
Resting heart rate (bpm)	64.207.32	59.676.28	62.336.91	1.658	0.203
Years of education (Y)	16.401.64	16.401.77	17.001.73	0.614	0.546

**IPSQ-S**					

High-intensity activity (min)	75.67134.39	182.33224.36	156.88325.48	0.789	0.461
Moderate-intensity activity (min)	62.6766.28	126.1591.34	77.1988.69	2.225	0.121
Walking activity (min)	324.00337.08	315.00261.30	257.19214.82	0.273	0.762

**Exercise intensity monitoring**					

Heart rate (bpm)	153.724.73	131.077.24	78.9118.35	68.36	<0.001
%HRR	68.272.45	52.554.32	12.5212.13	160.9	<0.001
RPE	16.411.68	14.082.07	8.252.12	216.3	<0.001

*Measurement data are expressed as mean ± standard deviation (X ± SD). BMI, body mass index; IPSQ-S, International Physical Activity Questionnaire–Short; RPE, rating of perceived exertion; %HRR, percentage of heart rate reserve.*

### Behavioral Results

#### Pressure Pain Threshold Results

**FIGURE 2:**
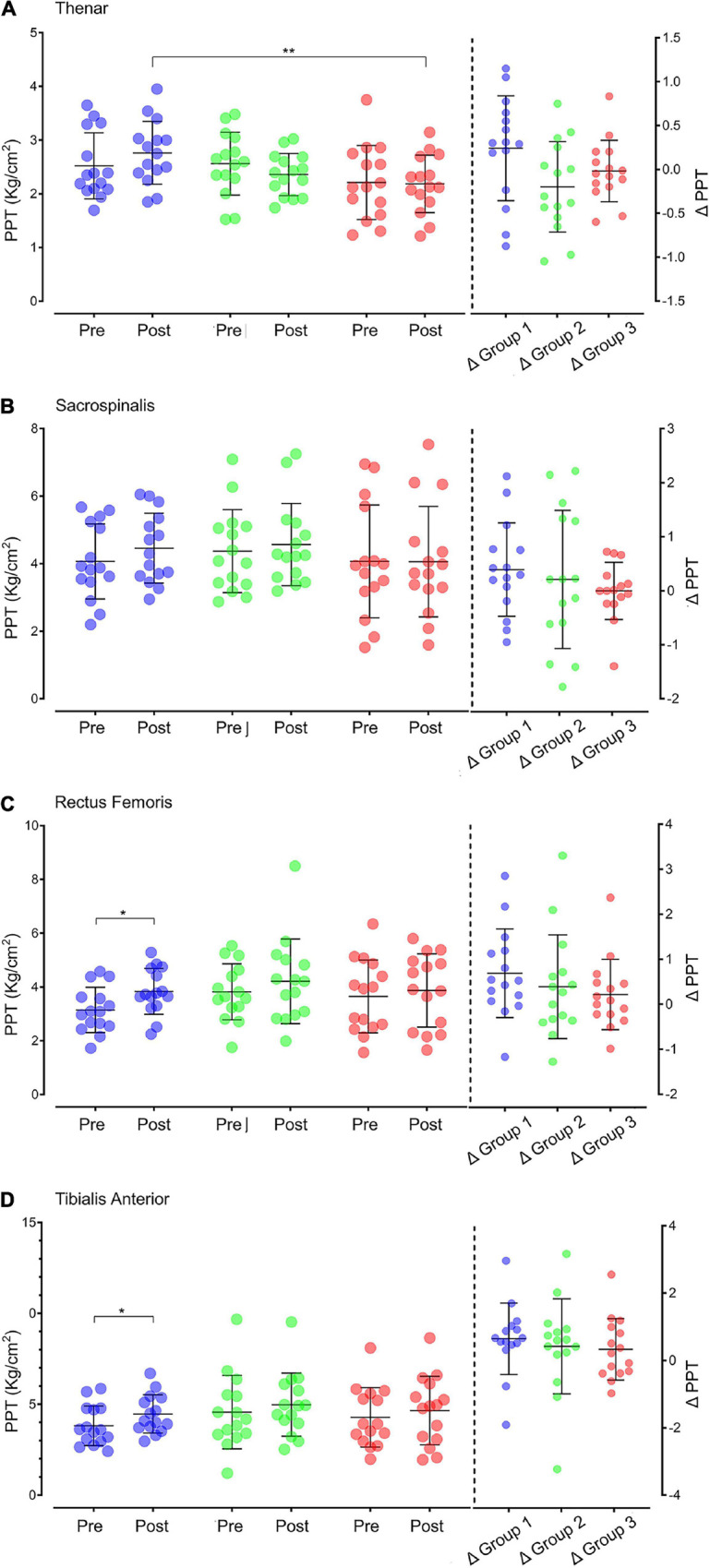
PPT in different sites before and after intervention in the three groups. **(A)** Thenar muscle PPT. **(B)** Sacrospinalis PPT. **(C)** Rectus femoris PPT. **(D)** Tibialis anterior PPT. On the left of the vertical dotted line are the PPT values before and after intervention of individuals (dots: purple for Group 1, green for Group 2, and red for Group 3) and groups (black line: X ± SD). The data on the right of the vertical dotted line are the individual (dot) and group (black line: X ± SD) values of the difference in PPT after intervention (△ = posttest–pretest). PPT: pressure pain threshold; Pre: pretest; Post: posttest. *p < 0.05; **p < 0.01.

The test results of PPT at different sites before and after intervention for the three groups are shown in Figure 2. No statistical difference in PPT existed among the groups before exercise. After intervention, a statistically significant difference in thenar PPT was observed among the three groups (*p* = 0.011). The difference between Groups 1 and 3 was the most statistically significant (*p* = 0.004). The difference in PPT before and after intervention was compared at the group level and found to be not statistically significant (*p* > 0.05).

The paired *t*-test showed that after intervention, the PPT of multiple sites in Group 1 increased significantly, including the PPT in rectus femoris (*p* = 0.017) and tibialis anterior muscle (*p* = 0.033).

#### Heat Pain Threshold Results

**FIGURE 3:**
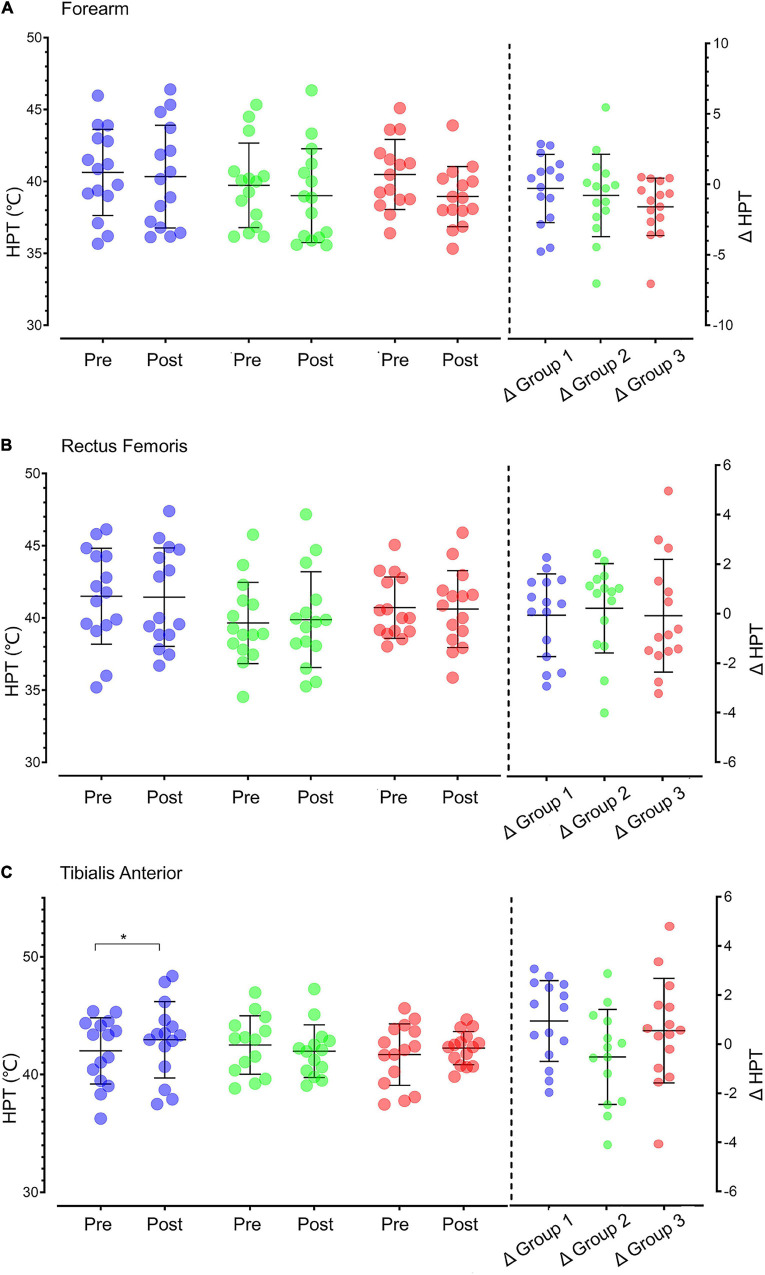
HPT in different sites in the three groups before and after intervention. **(A)** Forearm HPT. **(B)** Rectus femoris HPT. **(C)** Tibialis anterior HPT. The data on the left side of the vertical dotted line are the HPT values of individuals (dots: purple for Group 1, green for Group 2, and red for Group 3) and groups (black line: X ± SD) before and after intervention. The data on the right side of the vertical dotted line are the values of the difference in HPT after intervention (△ = posttest–pretest) for individuals (dot) and groups (black line: X ± SD). HPT, heat pain threshold; Pre, pretest; Post, posttest; *p < 0.05.

The results of HPT at different sites before and after intervention are shown in Figure 3. One-way ANOVA showed that no statistical difference in HPT in the forearm, rectus femoris and tibialis anterior muscle existed between groups before intervention, after intervention and their differences. The paired *t*-test revealed that the HPT of tibialis anterior muscle increased significantly after moderate-intensity aerobic exercise (*p* = 0.044).

#### Contact Heat Stimulus Results

**FIGURE 4:**
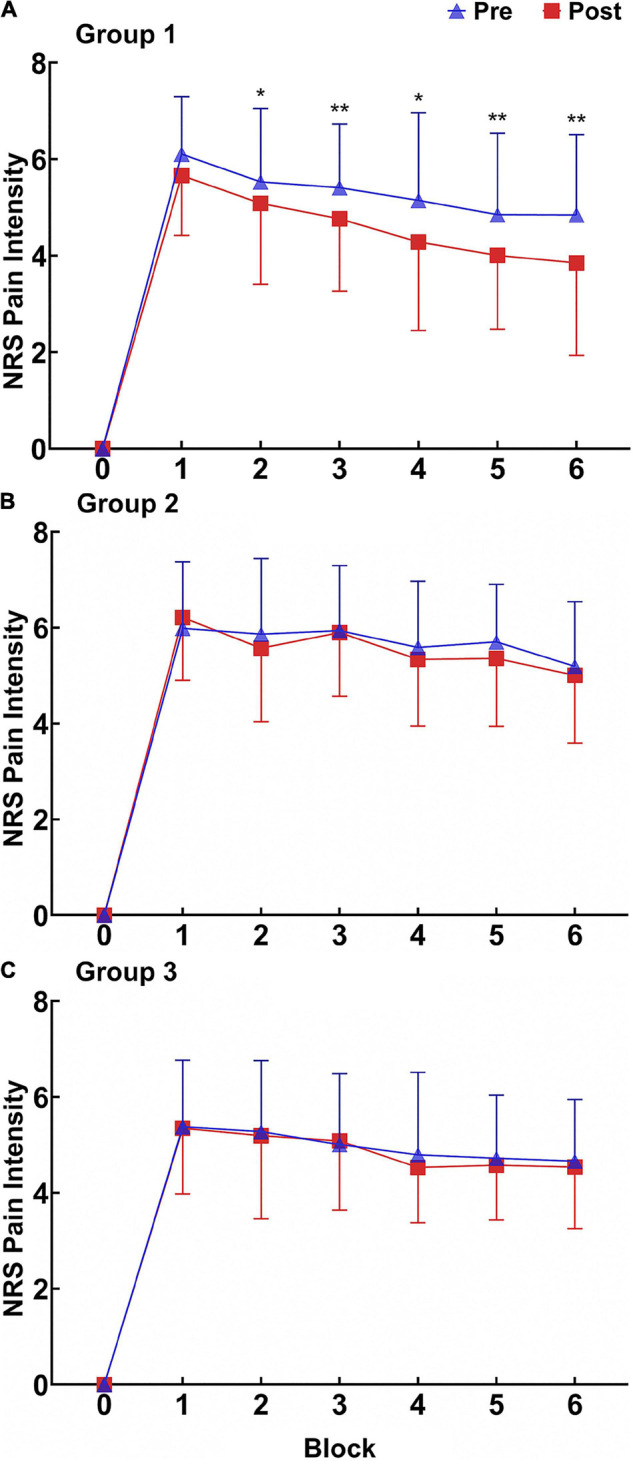
Pain intensity ratings (X ± SD) for RPHS before and after intervention in the three groups. **(A)** Group 1. **(B)** Group 2. **(C)** Group 3. NRS, numeric rating scales; Pre, pretest; Post posttest. *p < 0.05; **p < 0.01.

(1) Pain intensity ratings (Figure 4). At the intra-group level, the main effect of test time (pretest and posttest) was significant (*p* = 0.002), and the main effect of the block (Blocks 1–6) was significant (*p* < 0.001). At the intergroup level, the main effect of intervention conditions (moderate-intensity exercise, low-intensity exercise, and control) was not significant (*p* = 0.259). The interaction effect between test time and intervention conditions was significant (*p* = 0.024).

The paired *t*-test indicated that the pain intensity ratings of contact heat stimuli in Blocks 2, 3, 4, 5, and 6 decreased significantly in Group 1 (Block 2: *p* = 0.031; Block 3: *p* < 0.001; Block 4: *p* = 0.014; Block 5: *p* = 0.003; Block 6: *p* < 0.001). The difference between the posttest and pretest exhibited an increasing trend, suggesting that the hypoalgesic effect of moderate-intensity aerobic exercise still existed after the end of contact heat stimulus.

**FIGURE 5:**
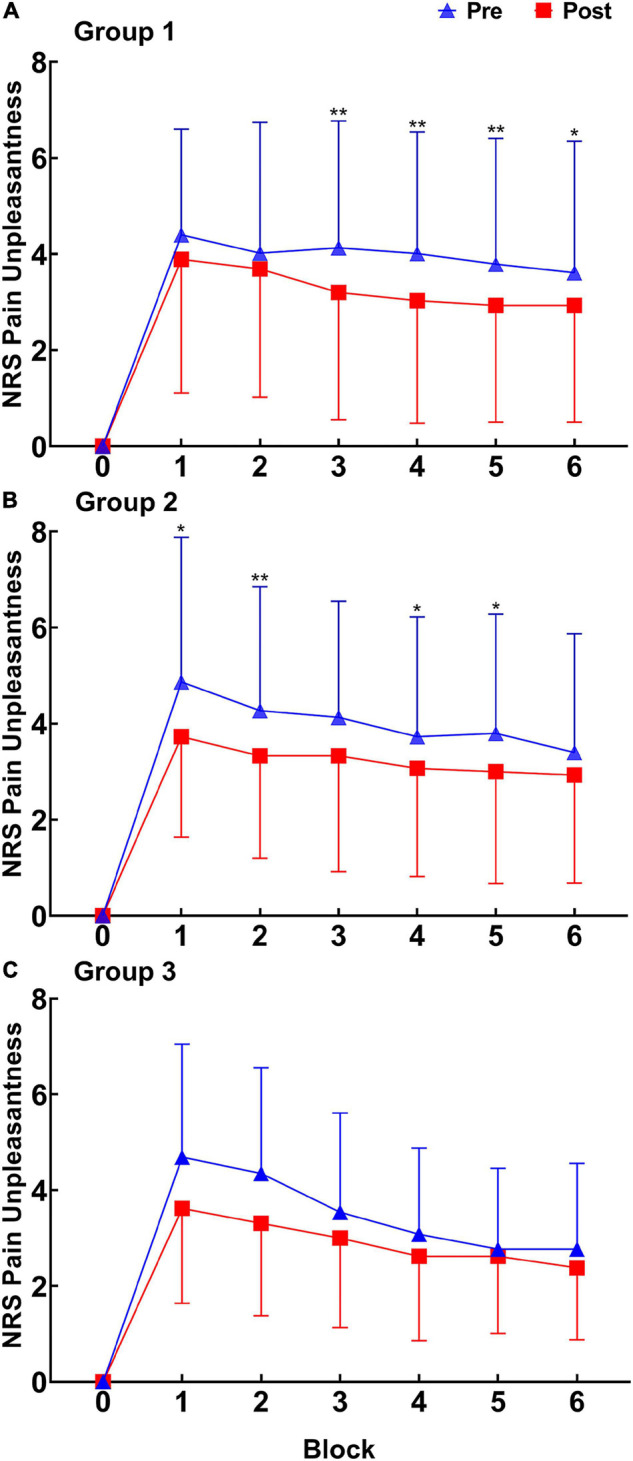
Pain unpleasantness ratings (X ± SD) of RPHS before and after intervention in the three groups. **(A)** Group 1. **(B)** Group 2. **(C)** Group 3. NRS, numeric rating scales; Pre, pretest; Post, posttest. *p < 0.05; **p < 0.01.

(2) Pain unpleasantness ratings (Figure 5). At the intra-group level, the main effects of test time and block were significant (*p* < 0.001 for test time and *p* < 0.001 for block). At the inter-group level, the main effect of intervention conditions was not significant (*p* = 0.856). The interaction effect between test time and intervention conditions was not significant (*p* = 0.874).

The paired *t*-test revealed that the pain unpleasantness ratings in Blocks 3, 4, 5, and 6 decreased significantly in Group 1 (Block 3: *p* < 0.001; Block 4: *p* = 0.005; Block 5: *p* = 0.003; Block 6: *p* = 0.010). The pain unpleasantness ratings in Blocks 1, 2, 4, and 5 decreased significantly in Group 2 (Block 1: *p* = 0.030; Block 2: *p* = 0.005; Block 4: *p* = 0.019; Block 5: *p* = 0.022). The difference between the posttest and pretest showed no increasing trend, suggesting that moderate-intensity aerobic exercise had different durations in regulating the pain emotional response and pain perception. Groups 1 and 2 had different blocks of regulating effects, suggesting that the regulation of the pain emotional response after the two aerobic exercises had a time difference.

### Event-Related Potential Results

#### Time-Domain Results

**FIGURE 6:**
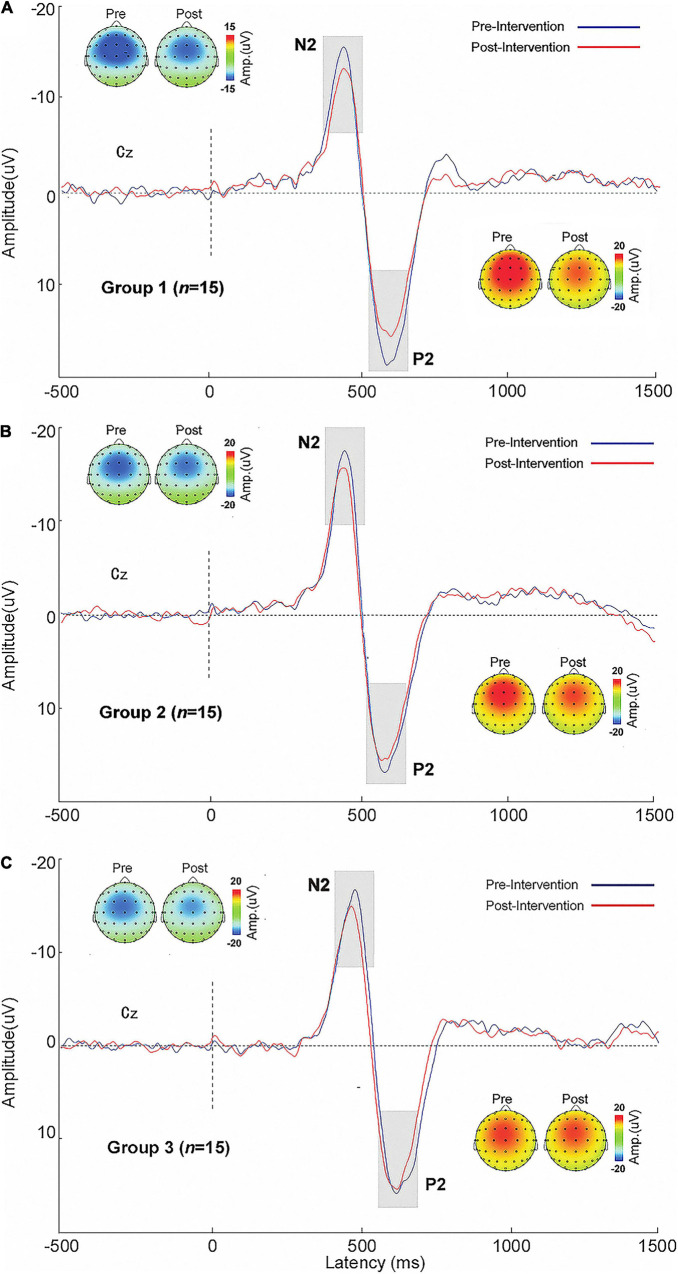
Contact heat-evoked potential waveforms and N2 and P2 topographic maps of the central parietal electrode (Cz) in the three groups before and after intervention. **(A)** Group 1. **(B)** Group 2. **(C)** Group 3. Pre, pretest; Post, posttest.

The ERP waveforms and topographic maps for the three groups before and after intervention are shown in Figure 6. The N2 and P2 amplitudes are presented in Table 2. No statistical significance existed in N2 and P2 amplitudes among the groups before and after intervention. At the intra-group level, the P2 amplitude of Group 1 after exercise was significantly lower than that before exercise (*p* < 0.05).

**TABLE 2:** N2 and P2 amplitudes before and after interventions [X ± SD (μV)].

		**Group 1 (*n* = 15)**	**Group 2 (*n* = 15)**	**Group 3 (*n* = 15)**	**F**	***p* ^†^**
N2	Pretest	17.56 ± 7.96	21.07 ± 7.04	21.07 ± 7.04	0.619	0.545
	Posttest	16.42 ± 8.39	19.32 ± 7.15	20.32 ± 9.30	0.614	0.548
	*p* ^‡^	0.296	0.105	0.887		
P2	Pretest	21.27 ± 9.95	20.55 ± 8.74	19.21 ± 8.20	0.136	0.874
	Posttest	18.56 ± 9.49	19.61 ± 8.00	19.14 ± 8.11	0.440	0.957
	*p* ^‡^	0.043^*^	0.229	0.914		

^*^p < 0.05; ^†^one-way ANOVA; ^‡^paired t-test.

#### Spectrum Results

**FIGURE 7:**
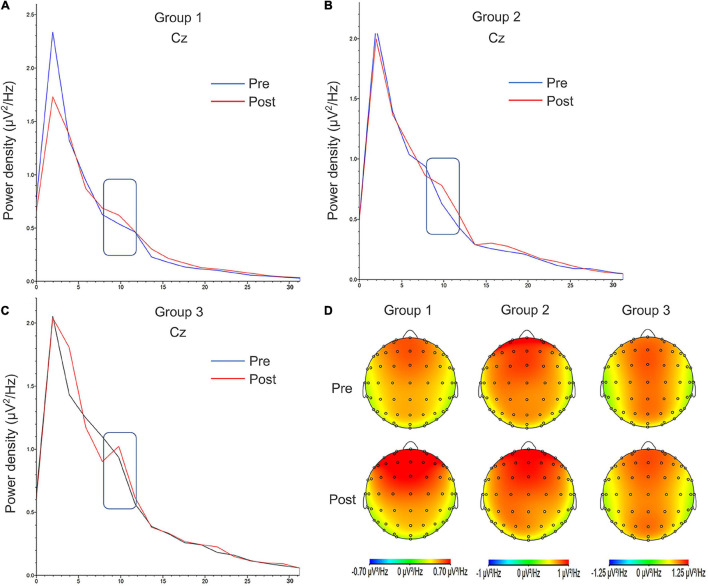
Power density distribution and alpha band topographic maps of the three groups before contact heat pain stimulation. **(A)** Group 1. **(B)** Group 2. **(C)** Group 3. **(D)** The topographic maps of alpha band oscillation power density before and after intervention. The alpha band (8–12Hz) is marked with a rectangular border. The alpha oscillation power density is largest in the frontal region. Pre, pretest; Post, posttest.

The change in alpha oscillation power is shown in Table 3. The power density distribution and alpha band topographic maps before and after contact heat stimuli are shown in Figure 7. At the intra-group and inter-group levels, no statistical difference in the power density of the alpha band existed before and after intervention. The paired *t*-test showed that the power density of the alpha band in Group 1 increased significantly after intervention (*p* = 0.046).

**TABLE 3:** Comparison of power density of alpha oscillation before and after intervention in three groups [X ± SD (μV^2^/Hz)].

		**Group 1 (*n* = 15)**	**Group 2 (*n* = 15)**	**Group 3 (*n* = 15)**	**F**	***p* ^†^**
Alpha oscillation (8–12Hz)	Pretest	0.55 ± 0.29	0.730.25	0.840.23	3.165	0.570
	Posttest	0.65 ± 0.31	0.720.23	0.830.18	1.320	0.282
	Posttest–pretest	0.10 ± 0.14	−0.010.15	−0.010.25	1.328	0.280
	*p* ^‡^	0.046^*^	0.867	0.745		

^*^p < 0.05; ^†^for one-way ANOVA; ^‡^paired t-test.

### Correlation Results

As shown in Table 4, a Spearman correlation analysis was conducted on the three types of exercise intensity index (RPE score, exercise heart rate, and %HRR) with a difference in the various indicators before and after intervention. The difference between pain intensity rating and %HRR showed a significant negative correlation (*r* = –0.438, *p* = 0.007). Alpha power density was significantly and positively correlated with RPE score and exercise heart rate (*r* = 0.372, *p* = 0.020; *r* = 0.374, *p* = 0.019). The difference between the PPT of the thenar muscle and exercise heart rate had a significant positive correlation (*r* = 0.403, *p* = 0.012). A significant positive correlation also existed between the PPT difference of the tibialis anterior muscle and %HRR (*r* = 0.386, *p* = 0.016).

**TABLE 4:** The correlation between the three exercise intensity indexes with the difference of various indicators before and after intervention.

	**RPE score**		**Exercise heart rate**		**%HRR**	
	**r**	***p***	**r**	***p***	**r**	***p***
Difference of pain intensity rating	–0.168	0.183	–0.196	0.145	–0.438	0.007**
Difference of pain unpleasantness rating	–0.282	0.065	–0.021	0.456	0.040	0.417
Difference of P2 amplitude	0.122	0.277	–0.040	0.423	–0.277	0.085
Difference of alpha oscillation power density	0.372	0.020*	0.374	0.019*	–0.119	0.262
Difference of forearm HPT	–0.120	0.260	–0.056	0.382	0.207	0.132
Difference of rectus femoris HPT	–0.107	0.283	–0.081	0.333	0.122	0.256
Difference of thenar muscle PPT	0.136	0.232	0.403	0.012*	0.151	0.209
Difference of sacrospinalis PPT	0.263	0.076	0.960	0.303	0.282	0.062
Difference of rectus femoris PPT	–0.022	0.453	0.025	0.448	0.185	0.159
Difference of tibialis anterior muscle PPT	0.054	0.386	0.156	0.201	0.386	0.016*

Difference = posttest–pretest; %HRR: percentage of heart rate reserve = (exercise heart rate—resting heart rate)/(220 - age—resting heart rate)^∗^100%. RPE, rating of perceived exertion; HPT, heat pain threshold; PPT, pressure pain threshold. *p < 0.05; **p < 0.01.

## Discussion

### Behavioral Evidence for Aerobic Exercise-Induced Hypoalgesia

The PPT results were consistent with those in previous studies. PPT and HPT increased after moderate-intensity aerobic exercise (Meeus et al., 2010; Kodesh and Weissman-Fogel, 2014), whereas low-intensity aerobic exercise had a limited effect on pressure pain sensitivity (Hoffman et al., 2004). The correlation analysis in this study indicated that the hypoalgesic effect of aerobic exercise was related to exercise intensity. The hypoalgesic effect may also be related to RPE score; thus, appropriate improvement of subjective fatigue within a certain range can enhance aerobic EIH (Peterson et al., 2019).

In terms of HPT, only the tibialis anterior muscle increased significantly after moderate-intensity aerobic exercise. No significant changes were observed in rectus femoris. Compared with mechanical stimulation, aerobic exercise exerted a limited influence on heat pain sensitivity (Jones et al., 2019). Previous research has shown that peripheral afference can partly explain EIH differences in different pain stimuli (de Souza et al., 2013). These differences are also related to the increase in skin temperature after aerobic exercise (Pertovaara et al., 1996). Likewise, an increase in local temperature can lead to heat pain sensitization. Cross-sectional studies have revealed that long-term vigorous physical exercise is associated with low pressure pain sensitivity and low heat pain sensitivity (Andrzejewski et al., 2010; Ellingson et al., 2012), which is considered to be the accumulation of the EIH effects of long-term exercise. Another possibility is that exercise exerts a greater impact on nociceptors located in the muscles than those in the skin. Pressure pain is likely to originate from deep nociceptors in the muscles, while heat pain is likely to originate from superficial nociceptors in the skin (Jones et al., 2019). Recent studies have found that blocking the blood flow of the limb during exercise can significantly reduce the EIH effect on the blocked limb (Jones et al., 2017). This condition may be related to the blocked transmission of hypoalgesic substances in the blood (Thorén et al., 1990). Further studies have shown that mechanical and heat pain are regulated by δ and μ opioid receptors, respectively (Scherrer et al., 2009). Meanwhile, cannabinoid receptors can regulate the input of mechanical and heat stimulation but have a greater impact on mechanical stimulation (Agarwal et al., 2007). Notably, a difference in test time increases the difference in test results. These explanations indicate that the differences in the EIH effects of different stimulus types still require further study.

In the present study, moderate-intensity aerobic exercise reduced the pain intensity and pain unpleasantness ratings in contact heat stimuli, whereas low-intensity aerobic exercise only influenced the pain unpleasantness ratings. These results indicate that the emotional response to pain is more affected by aerobic exercise than by pain perception. Some researchers used positron emission tomography to prove that pain stimulation, like hot water immersion stimulation, can selectively change the brain’s unpleasant response without changing the perception of pain intensity (Rainville et al., 1997). They also found that anterior cingulate gyrus neuron activity, encoding pain unpleasantness, is significantly changed, whereas the activation of the primary somatosensory cortex, encoding discriminative properties of somatosensory stimuli (i.e., intensity of noxious stimulation), remains unchanged; there seems to be at least a partial segregation of function between pain affect and sensation. These points were consistent with the findings of present study, for moderate-intensity aerobic exercise effectively reducing pain intensity and pain unpleasantness of contact heat stimulus and low-intensity aerobic exercise just selectively inhibiting the pain unpleasantness, for example.

The behavioral results of this study provided evidence that the EIH effect of moderate-intensity aerobic exercise is better than that of low-intensity aerobic exercise.

### Event-Related Potential Evidence for Aerobic Exercise-Induced Hypoalgesia

The hypoalgesic effect of aerobic exercise was further supported by the ERP results, which exhibited a significant difference in P2 amplitude before and after exercise. P2 is one of the main components of pain-evoked potential, and its amplitude is closely related to stimulus intensity and the subject’s pain perception (Iannetti et al., 2006; Huang et al., 2013). P2 mainly originates from the anterior and medial cingulate gyrus and reflect the cognitive and emotional processing of pain perception (Garcia-Larrea et al., 2003; Valentini et al., 2012).

The spectrum analysis results showed that after moderate-intensity aerobic exercise, alpha oscillation power was enhanced before contact heat stimulus. The correlation analysis indicated that alpha oscillation power was positively correlated with exercise intensity. Alpha oscillations before stimulation can regulate pain perception after stimulation (Haegens et al., 2011; Tu et al., 2016). Brain oscillation before stimulation reflects the central preparation for external stimulation and predicts the neural response caused by perception and subsequent stimulation (Laufs et al., 2003; Boly et al., 2007; Gilbert and Sigman, 2007). Studies have shown that alpha oscillation is an indicator of sensory cortical excitability and attention resource allocation of the somatosensory system (Haegens et al., 2011). In general, alpha oscillation before stimulation partly reflects the activation of the resting state sensory-motor neural network (Anderson and Ding, 2011; Weisz et al., 2014). In addition, alpha oscillation reflects the central descending inhibitory function (Mathewson et al., 2011; Klimesch, 2012). Therefore, this study indicated that the hypoalgesic mechanisms of aerobic exercise may involve the enhancement of the central descending inhibitory function for a significant increase in the power density of alpha oscillation after moderate intensity aerobic exercise.

This study has its limitations. First, the blood lactate threshold of the subjects was not measured in this study, and the subjects had different tolerance to exercise, which may have influenced the aerobic EIH efficiency. Second, the pain test indicators were limited, and the experimental pain caused by contact heat stimulus could not fully reflect the neurophysiological characteristics of daily musculoskeletal pain. Lastly, the EIH efficiency of low-intensity aerobic exercise in this work was insufficient and still needs to be evaluated comprehensively by increasing the number of exercise interventions. The correlation between pain sensitivity and psychological factors has drawn increasing attention to whether psychological factors mediate the effects of EIH, and this area requires further research.

In summary, aerobic exercise exerts an overall EIH effect. Its hypoalgesic effect is related to exercise intensity and is affected by the site and type of pain stimulus. Moderate-intensity aerobic exercise effectively reduces the pain sensitivity to various painful stimuli, and low-intensity aerobic exercise selectively inhibits the negative emotional pain response. An increase in alpha oscillation power before a stimulus indicates that the hypoalgesic mechanisms of aerobic exercise may involve the enhancement of the central descending inhibitory function. This study focuses on the neurophysiological mechanisms of the aerobic EIH effect of moderate- and low-intensity aerobic exercise. The findings can provide theoretical guidance for optimizing pain exercise prescription in clinical practice.

## Data Availability Statement

The raw data supporting the conclusions of this article will be made available by the authors, without undue reservation.

## Ethics Statement

The studies involving human participants were reviewed and approved by the Ethics Committee of Shanghai University of Sport. The patients/participants provided their written informed consent to participate in this study.

## Author Contributions

KZ, CC, SY, and XW conceptualized this study and contributed to revising and approving the final version of the manuscript. KZ and CC contributed to collecting data, analyzed the data, and drafted the manuscript. All authors contributed to the article and approved the submitted version.

## Conflict of Interest

The authors declare that the research was conducted in the absence of any commercial or financial relationships that could be construed as a potential conflict of interest.

## Publisher’s Note

All claims expressed in this article are solely those of the authors and do not necessarily represent those of their affiliated organizations, or those of the publisher, the editors and the reviewers. Any product that may be evaluated in this article, or claim that may be made by its manufacturer, is not guaranteed or endorsed by the publisher.
